# Diabetic Retinopathy Screening in Primary Care Real Practice: Study Procedures and Baseline Characteristics from the RETINAvalid Project

**DOI:** 10.3390/healthcare14030334

**Published:** 2026-01-28

**Authors:** Víctor-Miguel López-Lifante, Maria Palau-Antoja, Noemí Lamonja-Vicente, Cecilia Herrero-Alonso, Josefina Sala-Leal, Rosa García-Sierra, Adrià Prior-Rovira, Marina Alventosa-Zaidin, Meritxell Carmona-Cervelló, Erik Isusquiza Garcia, Idoia Besada, Pere Torán-Monserrat

**Affiliations:** 1 Unitat de Suport a la Recerca Metropolitana Nord, Institut Universitari d’Investigació en Atenció Primària Jordi Gol (IDIAP Jordi Gol), 08303 Mataró, Spain; mpalau@idiapjgol.org (M.P.-A.); nlamonjav.mn.ics@gencat.cat (N.L.-V.); cherreroal.mn.ics@gencat.cat (C.H.-A.); rgarciasi.mn.ics@gencat.cat (R.G.-S.); apriorr.mn.ics@gencat.cat (A.P.-R.); malventosa.bcn.ics@gencat.cat (M.A.-Z.); mcarmonace.mn.ics@gencat.cat (M.C.-C.); ptoran.bnm.ics@gencat.cat (P.T.-M.); 2 Gerència d’Atenció Primària Vallès Occidental i Vallès Oriental Institut Català de Salut, 08203 Sabadell, Spain; fsala.mn.ics@gencat.cat; 3 Palau-Solità Healthcare Centre, Palau-Solità Plegamans Institut Català de la Salut, 08184 Palau-Solità i Plegamans, Spain; 4 Department of Medicine, Faculty of Medicine, Universitat Autònoma de Barcelona, 08193 Bellaterra, Spain; 5 Germans Trias i Pujol Research Institute, Can Ruti Campus, Universitat Autònoma de Barcelona, 08916 Badalona, Spain; 6 Research Group on the Impact of Chronic Diseases and Their Trajectories, 08007 Barcelona, Spain; 7 Multidisciplinary Research Group in Health and Society GREMSAS, 08007 Barcelona, Spain; 8 Nursing Department, Faculty of Medicine, Universitat Autònoma de Barcelona, 08193 Bellaterra, Spain; 9 Gerència d’Atenció Primària Barcelonès Nord i Maresme, Institut Català de Salut, 08916 Mataró, Spain; 10 Arenys de Mar Healthcare Centre, Arenys de Mar Institut Català de la Salut, 08350 Arenys de Mar, Spain; 11 PhD Programme in Biomedical Research Methodology and Public Health, Universitat Autònoma de Barcelona, 08193 Bellaterra, Spain; 12 ULMA Medical Technologies S.L.U., 20560 Oñati, Spain; eisusquiza@ulma.com (E.I.G.); ibesada@ulma.com (I.B.); 13 Department of Medicine, Faculty of Medicine, Universitat de Girona, 17003 Girona, Spain

**Keywords:** diabetic retinopathy, screening, retinography, primary care, artificial intelligence

## Abstract

**Background/Objectives**: With rising diabetes rates, early detection of complications such as diabetic retinopathy (DR), a leading cause of visual impairment, is crucial. Incorporating DR screening into primary care has shown positive results, and integrating technological advances and artificial intelligence (AI) into these processes offers promising potential. The overall study aims to evaluate the agreement between primary care physicians, ophthalmologists, and an AI system in DR screening and referral decisions within a real-world primary care setting. **Methods**: In this brief report, we present the study protocol and provide an initial overview and description of our sample. A total of 1517 retinographies, obtained by a non-mydriatic retinal camera, were retrospectively collected from 301 patients with diabetes. **Results**: Primary care physicians referred 34.5% of the patients to ophthalmology, primarily due to opacification, suspicion of DR, or other retinal diseases. Overall, 13.62% of the participants were suspected of having DR, with 9.63% having a definitive diagnosis. **Conclusions**: These initial descriptive findings will be further explored in the next phase of the study through the analysis of concordance between primary care physicians, the AI-based software, and ophthalmology specialists. Future results are expected to provide valuable insights into the reliability of DR screening across different evaluators and support the integration of effective DR screening strategies into real-world clinical practice.

## 1. Introduction

Diabetes is a chronic metabolic disorder characterized by elevated blood glucose levels resulting from insulin deficiency, insulin resistance, or a combination of both [1,2]. It affects 589 million adults worldwide (4.7 million in Spain), representing 11.1% of the population aged 20–79 [3]. Type 2 diabetes (T2D) accounts for over 90% of all cases and is primarily associated with peripheral insulin resistance and a progressive loss of adequate insulin secretion, whereas type 1 diabetes (T1D) is an autoimmune β-cell destruction that leads to absolute insulin deficiency [4].

Diabetes causes multiple complications, affecting many organ systems. These chronic complications develop as a result of hyperglycemia, dyslipidemia and alterations in other metabolic pathways, and are responsible for most of the mortality attributed to the disease, which was estimated at 3.4 million deaths in 2024 [3,5]. Common complications include cardiovascular disease, kidney disease, neuropathy, retinopathy, and lower-limb amputation, as well as other emerging and less acknowledged complications [5,6,7].

The ageing of the world’s population, urbanization, and lifestyle changes are significantly contributing to the rising prevalence of diabetes observed in recent decades, and the number of adults living with this condition is expected to reach 853 million by 2050 [3]. This increasing trend, the number of people living with undiagnosed diabetes, and the growing prevalence of prediabetes pose significant healthcare challenges and a substantial burden on public health systems worldwide, underscoring the need for prevention, early diagnosis, monitoring, and treatment to effectively manage diabetes and its associated complications and improve patients’ quality of life [2,3,8].

Diabetic retinopathy (DR) is the major ocular complication of diabetes and a leading cause of blindness and visual impairment [9]. Around 30% of people with diabetes exhibit signs of DR, and a proportion below 10% presents sight-threatening lesions, although this varies depending on the region and type of diabetes [3,10]. In Spain, the current prevalence of DR in type 2 diabetes patients is 15.28% and 1.92% for sight-threatening diabetic retinopathy (STDR), with an annual incidence of 3.83% and 0.41%, respectively [11].

DR is a microangiopathy in which persistent high blood glucose levels result in damage to the blood vessels of the retina [9]. The most relevant risk factors for DR are poor glycemic control, years of evolution of diabetes, and hypertension, although the development and progression of DR cannot be solely explained by known risk factors, and genetic determinants and glucose variability may also play a role in it [12,13].

Diabetic retinopathy is classified into non-proliferative diabetic retinopathy (NPDR) and proliferative diabetic retinopathy (PDR), including five stages of severity: no apparent retinopathy, mild NPDR, moderate NPDR, severe NPDR, and PDR [14].

A common complication of DR that can occur at any stage of the disease is diabetic macular edema (DME), which is defined as thickening of the retina and can result in vision loss [9,15]. DME is classified regarding the absence or presence of retinal thickening or lipid in the posterior pole, with three stages of severity [14]:

1. Mild DME, characterized by some retinal thickening or hard exudates distant from the center of the macula.
2. Moderate DME, characterized by retinal thickening or hard exudates approaching the center of the macula but not involving the center.
3. Severe DME, characterized by retinal thickening or hard exudates involving the center of the macula.

Patients with DR can remain asymptomatic until the disease is advanced, at which point the likelihood of effective treatment is considerably reduced [9,15]. Therefore, screening, surveillance, and early detection are of vital importance to prevent or delay the onset or progression of the disease and reduce the risk of vision impairment. Moreover, DR screening can expand beyond the prevention and diagnosis of sight-threatening disease and help to identify patients at risk of other complications, such as cardiovascular disease and cognitive impairment [13]. Current guidelines recommend screening within five years after the onset of diabetes for T1D patients and at the time of diagnosis for T2D patients, and repeated every 1–2 years [16].

Retinal examination requires retinography or fundus photography, which must be performed and interpreted by well-trained personnel to ensure optimal referral and management [9,12].

Primary care physicians play a pivotal role in the management of diabetes and its associated complications, including diabetic retinopathy [15,17]. DR screening has been implemented in primary care settings for several years, increasing coverage and accessibility [17,18]. The inclusion of general practitioners in the screening processes has shown positive results, although specific training is needed to ensure proper retinography interpretation and referral to ophthalmology [17,18,19,20,21].

Technological advancements have positioned telemedicine as a valuable tool to enhance access to screening, and facilitate timely evaluations, referrals, and follow-up. This approach has emerged as a cost-effective strategy for optimizing healthcare professionals’ time management and workload [22]. Furthermore, artificial intelligence (AI) is bound to play an important role in upcoming years by assisting with diagnosis, screening and prognostication, thereby enabling personalized medical care while reducing the burden on healthcare professionals [9,23]. The development of AI-based systems has significantly advanced DR screening, demonstrating reliable diagnostic performance which has led to their approval for clinical use and implementation in primary care settings [9,10,13,23,24,25].

The integration of AI into screening workflows within primary care settings has the potential to standardize diagnostic processes and significantly enhance practice efficiency, reducing the workload for both ophthalmology specialists and primary care physicians, ultimately leading to improved patient care [23,24].

This study aims to evaluate the adequacy of diabetic retinopathy screening through retinography in a real-world primary healthcare setting by assessing the concordance between primary care and specialist diagnoses and the appropriateness of referrals to ophthalmology. Additionally, it will examine the usefulness and effectiveness of an AI-based system for automated retinography reading by evaluating its diagnostic agreement with specialist assessments, with the goal of determining its potential to support clinical decision-making and streamline workloads.

## 2. Materials and Methods

### 2.1. Study Design

The RETINAvalid Study is a retrospective study conducted in the Northern Metropolitan Area of Barcelona (Spain) evaluating inter-observer concordance between the primary care physician’s initial diagnosis in a population-based DR screening by retinography and the reassessment of an ophthalmology specialist and an AI software. The study area corresponds to a total source population of 2,108,882 inhabitants. This study is being conducted in accordance with the Declaration of Helsinki and was approved by the ethics committee of the Foundation University Institute for Primary Health Care Research Jordi Gol i Gurina (ref. 21/108-PCV).

### 2.2. Participants

Adult individuals (i.e., ≥18 years old) diagnosed with T1D or T2D from two Primary Health Care (PHC) centers in the Northern Metropolitan Area of Barcelona, serving a total of 31,358 registered individuals (15,747 and 15,611, respectively), were invited to participate in the study via telephone between 22 November 2021 and 5 September 2022. Interested subjects were scheduled for an in-person appointment to provide further information about the study and obtain their informed consent. Inclusion criteria required participants to have undergone a screening retinography within the previous six years.

Patients suffering from dementia or other medical conditions affecting their decision-making capacity, as well as patients with pathologies or history of conditions that can affect the quality of the retinography, such as movement disorders or conditions causing opacification of the cornea, lens, or vitreous humor, including cataracts, leukoma, high myopia, vitreous degeneration or hemorrhage, corneal cicatrix, monocular vision, and retinal surgery, were excluded from the study.

### 2.3. Data Collection and Variables

Sociodemographic and clinical data were obtained from the participants’ medical records. Sociodemographic variables encompassed age—which was subsequently categorized into four groups, 45–54, 55–64, 65–75, and >75—and sex. Clinical variables focused on aspects related to diabetes and metabolic control. These include diabetes type, year of diagnosis, and treatment, comprising pharmacological and non-pharmacological approaches, as well as any intervention targeting the retina. Metabolic control was assessed using the most recent available measurement of glycated hemoglobin (HbA1c) levels.

Cardiovascular risk was also evaluated by collecting the closest available measurements to the retinography of cholesterol levels, systolic and diastolic blood pressure, ankle–brachial index, and abdominal perimeter. A variety of related comorbidities were also obtained from the participants’ medical records, such as hypertension (I10) and coronary disease, including angina (I20), myocardial infarction (I25.2), and chronic ischemic heart disease (I25.5). The subject’s smoking status was classified as non-smoker (never smoked), former smoker (at least 1 year without smoking), or current smoker.

The screening retinographies of the previous six years were obtained from the participants’ clinical histories. All images had been acquired by an experienced technician using a TOPCON TRC-NW6S, Topcon Europe Medical B.V., Sant Just Desvern, Spain, non-mydriatic retinal camera during an opportunistic screening in a primary care setting. Pharmacological mydriasis had been performed if necessary to obtain adequate image quality. Along with the retinal images, the corresponding retinography reports were also extracted. These reports included the date of image acquisition, the assessment made by the primary care physician regarding the presence of retinal lesions suggestive of DR, whether a referral to ophthalmology was made and the reason for it, as well as the ophthalmology report with the final diagnosis and classification of DR, if applicable.

### 2.4. Ophthalmology Specialist and AI Software Assessments

To ensure patient confidentiality, all retinographies were anonymized through a recodification process, in which each image was assigned a unique alphanumeric code that replaced any personally identifiable information.

In subsequent phases of the study, the retinographies will be evaluated by two ophthalmology specialists, who will participate in a training session to become acquainted with the platform containing the images and to unify the criteria applied in their annotations and assessments. The ophthalmologists will be aware that all images come from diabetic patients who were part of a screening strategy for DR within a primary care practice, but will have no access to the retinography reports, the diagnosis, or the patient’s characteristics.

The specialist assessment will entail a valuation of the retinal image quality, categorized as good, fair, or poor, as well as the identification of key anatomical landmarks, including the fovea and macula. They will be asked to identify the presence of injuries indicative of DR, such as microaneurysms, retinal hemorrhages, hard or soft exudates, intraretinal microvascular abnormalities, venous beading, neovascularization and vitreous or preretinal hemorrhages, specifying their location and extent. All retinal images will be classified by the ophthalmologists into one of the five recognized DR stages (no DR, mild NPDR, moderate NPDR, severe NPDR, or PDR), as well as into one of the DME stages (no DME, mild DME, moderate DME, or severe DME).

In case of disagreement between the two specialists regarding the interpretation of the retinography, a third ophthalmologist will independently review the image to provide a final assessment, establishing the gold standard diagnosis.

The AI assessment will be performed using UMI DR v1.0.0, Ulma Medical technologies S.L. Oñati, Spain, an AI-based software developed by ULMA, with the goal of providing a reliable diagnostic tool for automatic DR screening to improve coverage, efficiency, and early detection and diagnosis of diabetic retinopathy in primary care centers [26]. Using the ophthalmologist’s assessment as the reference standard, the UMI DR system has demonstrated high diagnostic performance in previous validation studies, achieving a sensitivity of 95.69% (95% CI: 90.23–98.59) and a specificity of 94.44% (95% CI: 93.52–95.31) for the detection of more than mild diabetic retinopathy. UMI DR is a CE-marked medical device certified in accordance with Regulation (EU) 2017/745 (MDR) following conformity assessment by Notified Body 0051. The AI assessment will involve the automated analysis of fundus images to determine the presence of moderate NPDR or more severe stages, allowing the system to classify whether the patients should continue routine monitoring in primary health care or to refer the patient to a specialist.

No additional interventions on the patients will be required, as all data were collected from existing medical records. In the event of identifying patients who had not undergone a retinography in accordance with clinical best practices, they will be referred to their primary care team to ensure adherence to recommended screening protocols and appropriate follow-up.

### 2.5. Sample Size

Assuming a diabetic retinopathy prevalence of 60% among patients enrolled in screening programs and a minimum acceptable Cohen’s kappa coefficient (κ) of 0.55, a sample size of 625 retinographies is required to detect a true κ value of at least 0.643, with a statistical power of 80% and a significance level of 5%. This sample size calculation was performed to justify planned concordance analyses in future phases of the study, while this report is limited to descriptive baseline data and does not include concordance analyses at this stage.

### 2.6. Statistical Analysis

Descriptive analysis used absolute frequencies and percentages for categorical variables, and mean and standard deviation for quantitative variables.

In order to assess the diagnostic performance of the primary care physician in a real-world practice setting, we will compare their initial assessments with the specialist reevaluations, which will be defined as the gold standard. Diagnostic performance will be quantified by calculating sensitivity, specificity, and accuracy. Additionally, inter-rater agreement between the primary care physician and the specialist will be analyzed using Cohen’s kappa coefficient (κ), with values ranging from 0 to 1, conventionally interpreted as slight (κ ≤ 0.20), fair (κ 0.21–0.40), moderate (κ 0.41–0.60), substantial (κ 0.61–0.80), and almost perfect (κ > 0.80) [27].

The diagnostic performance of the AI system will also be evaluated by comparing its assessments to those of the specialists. Sensitivity, specificity, and accuracy, along with inter-rater agreement, will be calculated.

Statistical significance will be assumed as *p* < 0.05 for all tests, and 95% confidence intervals (CI) will be reported. All analyses will be performed using R version 4.4.0.

## 3. Results

This report presents the preliminary results of our study, providing an initial overview and description of our sample.

### 3.1. Participants’ Characteristics

From an initial sample of 329 individuals, a total of 301 (91.5%) subjects were included in the study. Altogether, 1517 retinographies were collected, corresponding to an average of 4.67 (±2.15) images per patient in the six-year study period. Sociodemographic and clinical characteristics of the participants are shown in Table 1.

### 3.2. Screening and Referral to Ophthalmology

According to patients’ medical records, 29 (9.63%) of the participants were diagnosed with diabetic retinopathy. Screening retinography readings led primary care physicians to refer 34.5% of the patients to ophthalmology. The reasons for referral are shown in Table 2.

Overall, diabetic retinopathy was suspected in 13.62% of the patients included in the study, with nine participants exhibiting additional indications for referral beyond DR suspicion.

## 4. Discussion

The aim of the RETINAvalid Study is to evaluate the inter-observer concordance between primary care physicians, ophthalmology specialists, and an AI software in a population-based screening for diabetic retinopathy, specifically assessing the adequacy of patient referrals to ophthalmology based on retinography images obtained by a non-mydriatic retinal camera in a primary healthcare setting. In this brief report, we presented the preliminary results of the study, providing a descriptive analysis of the sociodemographic and clinical characteristics of our sample and an overview of the referrals to ophthalmology by primary care physicians.

Our study population consisted predominantly of older adults (mean age of 70.3 years old) with type 2 diabetes, reflecting both the higher prevalence of diabetes in this age group and the growing challenges aging societies face [3,28]. Women accounted for 44.5% of the cases, underscoring the relevance of considering sex and age-related differences in diabetes prevalence and outcomes to provide personalized diabetes care that suit individual patient profiles [29,30,31].

Participants in our study had a mean diabetes duration of 12.8 years, a known risk factor for DR development and progression [32,33]. The average level of glycated hemoglobin was 7.4%, slightly above the standard recommended target of <7.0%, but acceptable given the advanced age of the sample and the need for individualized glycemic goals [28,34].

Prior research highlights the complex interplay of risk factors shared by diabetes and diabetic retinopathy. Hypertension was highly prevalent in our sample, affecting 74.7% of the participants, which is consistent with previous reports showing a 50–80% prevalence among individuals with type 2 diabetes [35,36]. Elevated blood pressure is a risk factor for both diabetes and diabetic retinopathy, highlighting the importance of blood pressure and glycemic control not only to limit DR progression but also cardiovascular complications [12,13,35,36]. Notably, mean blood pressure in our sample (131.5/75.9 mmHg) aligns with the recommended target of <140/90 mmHg for this population [37].

Similarly, dyslipidemia and hypercholesterolemia are common comorbidities of T2D and have been associated with retinopathy and its progression, as elevated cholesterol and triglyceride levels contribute to retinal damage [38,39]. In our sample, however, total cholesterol and LDL-cholesterol levels remained within acceptable ranges.

Diabetes significantly increases the risk of cardiovascular complications, a leading cause of morbidity and mortality associated with the disease, with coronary disease representing a major burden and affecting patients’ quality of life [6,40]. In our study, 12.3% of participants were diagnosed with coronary disease. Cardiovascular risk in diabetes is shaped by multiple shared factors, including hypertension, hypercholesterolemia, microvascular complications, poor glycemic control, age, sex, body mass index, and lifestyle behaviors [6,40]. Notably, diabetic retinopathy has been linked to cardiac changes and a high prevalence of these risk factors, supporting its potential role as a predictor of coronary artery disease and highlighting the value of routine DR screening for broader insights into patients’ cardiovascular risk profiles [41]. Diabetes is also a well-established risk factor for peripheral artery disease. While the ankle–brachial index values in our sample suggested an absence of significant disease, its association with DR, and particularly PDR, puts emphasis on the importance of early screening to prevent vascular complications [42].

Given the rising global prevalence of both diabetes and obesity, understanding their close pathophysiological relationship is crucial [43]. Shared mechanisms between these conditions increase the risk of cardiovascular and other diabetes-related complications [44,45]. The observed mean abdominal perimeter of 103.8 cm, a proxy for central obesity, indicates elevated cardiovascular risk, while both waist circumference and body mass index have been identified as potential risk factors for diabetic microvascular complications, including DR [45]. These findings underscore the importance of weight management and targeted obesity interventions in patients with diabetes.

Collectively, these insights reinforce the multifactorial nature of diabetes and its complications, with multiple interrelated risk factors requiring targeted control to optimize patient health and quality of life. An integrated and multidisciplinary approach to diabetes is therefore essential to improve glycemic control, overall health, and long-term outcomes.

Lifestyle factors play a pivotal role in the development of type 2 diabetes, making lifestyle modification a cornerstone of diabetes management, treatment, and prevention [6]. While most participants were non-smokers (55.8%), tobacco use is associated with an increased risk of pre-diabetes and diabetes, poor glycemic control, and insulin resistance [46,47]. Smoking also contributes to higher mortality and cardiovascular risk in patients with diabetes, though its impact on DR still remains unclear [46,47].

Diet and physical activity are central pillars of the non-pharmacological approach of diabetes treatment, with established benefits for glycemic control, insulin sensitivity, cardiometabolic outcomes, and overall health [6,48,49]. In our sample, 71.4% of participants received dietary recommendations, underscoring diet as a key modifiable factor in diabetes care. When lifestyle and psychosocial interventions are insufficient, pharmacological treatment is necessary, reinforcing the value of a holistic approach to diabetes care [6]. With the goal of achieving and maintaining normal glycemic levels, patients in our study were primarily treated with metformin, insulin, DPP-4 inhibitors, sulfonylureas, and glucosuric agents.

With diabetic retinopathy being a leading cause of preventable blindness screening has become crucial for timely detection and intervention. In our study, 34.5% of patients were referred by primary care physicians to ophthalmology, mainly because of opacification, suspicion of DR, or other retinal diseases. Our results align with the findings of other studies, in which referral rates go from 9.59% to 56.8% [19,21,50,51,52,53,54,55]. This highlights the value of screening retinographies not only for detecting DR but also for identifying other retinal pathologies or conditions. This broader diagnostic scope, along with the utility of retinography screening in identifying patients at risk for other diabetes-related complications, highlights the potential additional benefits patients may gain from regular screening.

At the same time, referring only about one third of the patients contributes to alleviating the workload of specialists, thereby allowing them to prioritize and focus their attention on patients requiring ophthalmic care. Moreover, incorporating DR screening into primary care settings improve accessibility and coverage by enabling early detection within the patient’s usual healthcare environment, while engaging general practitioners in this process enhances their diagnostic capabilities and contributes to better patient care and monitoring [17,18,21,56].

In our study, 13.62% of patients screened were suspected of having DR, and 9.63% received a definitive diagnosis. These prevalence rates are consistent with other studies conducted in Spain [11,19,21,50,51,52,53], supporting the reliability of our primary care screening approach. Furthermore, 82.7% (24/29) of patients diagnosed with DR were referred to ophthalmology by primary care physicians, indicating effective identification and referral within the primary care setting. However, these preliminary results require further exploration with the analysis of the concordance between primary care physicians and ophthalmology specialists regarding referral decisions and their adequacy, which will be the focus of the next phases of our study.

Despite these positive preliminary results, accurate detection remains essential to ensure timely management and prevent vision loss, guaranteeing that all patients with DR receive appropriate specialist care. To address potential gaps in referral pathways, objective retinography interpretation, particularly under real-world clinical practice conditions, is essential to enhance diagnostic consistency and optimize referral decisions [57].

In this context, the introduction of telemedicine into screening processes has helped meet this challenge by facilitating enhanced follow-up and achieving high patient satisfaction, while reducing the burden on primary care providers [52,56]. Moreover, and even though barriers to implementation still exist, AI holds vast potential to further alleviate the workload of primary care physicians and improve not only diagnostic accuracy but also various aspects of personalized health care, significantly improving patient outcomes globally [56,58,59]. Accordingly, a key step in our study will focus on the analysis of the concordance between an AI-based software and the ophthalmologists’ assessments to evaluate its effectiveness as a supportive tool in DR screening.

### Limitations

The study presents several limitations that should be acknowledged. First, the results presented in this report are preliminary, and definitive conclusions cannot be drawn until concordance analyses are performed. Second, the retrospective nature of the study and the requirement for a retinography within the previous six years may introduce selection bias toward patients who are more engaged with healthcare systems. Third, the study population consists almost entirely of older adults with type 2 diabetes from a single geographical region in Spain, which may limit the extrapolation of the findings to other populations or healthcare systems. Future research should aim to include a more diverse and representative sample to ensure the generalizability of the findings.

## 5. Conclusions

The preliminary descriptive results of our study suggest that diabetic retinopathy screening using a non-mydriatic retinal camera in primary care leads to referral of approximately one third of patients to ophthalmology, while also enabling the detection of other retinal conditions requiring specialist follow-up. These findings will be further explored through the analysis of concordance between primary care physicians, an AI-based system, and ophthalmology specialists, with the aim of contributing to the enhancement of screening processes and ultimately improving patient care and outcomes.

## Figures and Tables

**Table 1:** Sociodemographic and clinical characteristics of the participants.

	Participants (*n* = 301)
**Sociodemographic characteristics**	
Sex, women, *n (%)*	134 (44.5)
Age, years, *mean (SD)*	70.3 (±9.67)
Age group, *n (%)*	
45–54	17 (5.65)
55–64	61 (20.3)
65–74	103 (34.2)
≥75	120 (39.9)
**Clinical characteristics: diabetes and metabolic control**	
Diabetes type, *n (%)*	
T1D	2 (0.66)
T2D	299 (99.3)
Duration of disease, *mean (SD)*	12.8 (±5.84)
Glycated hemoglobin, %, *mean (SD)*	7.40 (±1.40)
Treatment ^1^, *n (%)*	
Diet	215 (71.4)
Metformin	234 (77.7)
Insulin	94 (31.2)
DPP-4 inhibitors	83 (27.6)
Sulfonylureas	54 (17.9)
Glucosuric antidiabetic	42 (13.9)
Glinides	23 (7.64)
Alpha-glucosidase inhibitors	5 (1.66)
Glitazones	4 (1.33)
GLP-1 agonists	3 (1.00)
**Clinical characteristics: associated risk factors**	
Comorbidities, *n (%)*	
Hypertension	225 (74.7)
Coronary disease	37 (12.3)
Blood pressure, mmHg, *mean (SD)*	
Systolic blood pressure	131.5 (±12.4)
Diastolic blood pressure	75.9 (±8.28)
Cholesterol, mg/dL, *mean (SD)*	
Total cholesterol	183.9 (±39.6)
LDL cholesterol	105.0 (±34.4)
Ankle–brachial index, *mean (SD)*	
Right	1.13 (±0.12)
Left	1.13 (±0.10)
Abdominal perimeter, cm, *mean (SD)*	103.8 (±12.6)
Smoking status, *n (%)*	
Non-smoker	168 (55.8)
Former smoker	98 (32.6)
Smoker	35 (11.6)

^1^ Subjects may be under more than one treatment option.

**Table 2:** Reasons for referral to ophthalmology by primary care physicians and DR diagnosis.

Reason(s) for Referral, *n* (%)	Diagnosis of Diabetic Retinopathy	
	No (*n* = 272)	Yes (*n* = 29)
No referral	192 (70.6)	5 (17.2)
Suspicion of diabetic retinopathy	15 (5.51)	17 (58.6)
Age-related macular degeneration	1 (0.37)	0 (0.00)
Macular edema	1 (0.37)	0 (0.00)
Opacification	40 (14.7)	3 (1.10)
Other retinal diseases	14 (5.15)	0 (0.00)
More than one reason	9 (3.31)	4 (1.47)

## Data Availability

The data presented in this report is part of an ongoing study and is only available to the participating researchers due to privacy and ethical restrictions.

## Abbreviations

The following abbreviations are used in this manuscript:

DR	Diabetic Retinopathy
AI	Artificial Intelligence
T2D	Type 2 Diabetes
T1D	Type 1 Diabetes
NPDR	Non-proliferative Diabetic Retinopathy
PDR	Proliferative Diabetic Retinopathy
DME	Diabetic Macular Edema
PHC	Primary Health Care
